# Nonsurgical therapy for hydrocephalus: a comprehensive and critical review

**DOI:** 10.1186/s12987-016-0025-2

**Published:** 2016-02-05

**Authors:** Marc R. Del Bigio, Domenico L. Di Curzio

**Affiliations:** 1 Department of Pathology, University of Manitoba; Children’s Hospital Research Institute of Manitoba, Diagnostic Services Manitoba, 401 Brodie Centre, 715 McDermot Avenue, Winnipeg, MB R3E 3P5 Canada; 2 Department of Human Anatomy and Cell Science, University of Manitoba, Winnipeg, Canada

**Keywords:** Brain, Cerebrospinal fluid, Clinical trials, Diuretic, Drug therapy, Fibrinolysis, Hydrocephalus, Inflammation, Intracranial hemorrhage, Neuroprotective

## Abstract

Pharmacological interventions have been tested experimentally and clinically to prevent hydrocephalus and avoid the need for shunting beginning in the 1950s. Clinical trials of varied quality have not demonstrated lasting and convincing protective effects through manipulation of cerebrospinal fluid production, diuresis, blood clot fibrinolysis, or manipulation of fibrosis in the subarachnoid compartment, although there remains some promise in the latter areas. Acetazolamide bolus seems to be useful for predicting shunt response in adults with hydrocephalus. Neuroprotection in the situation of established hydrocephalus has been tested experimentally beginning more recently. Therapies designed to modify blood flow or pulsation, reduce inflammation, reduce oxidative damage, or protect neurons are so far of limited success; more experimental work is needed in these areas. As has been recommended for preclinical studies in stroke and brain trauma, stringent conditions should be met for preclinical studies in hydrocephalus.

**Electronic supplementary material:**

The online version of this article (doi:10.1186/s12987-016-0025-2) contains supplementary material, which is available to authorized users.

## Background

Hydrocephalus was defined by Rekate as “an active distension of the ventricular system of the brain resulting from inadequate passage of cerebrospinal fluid (CSF) from its point of production within the cerebral ventricles to its point of absorption into the systemic circulation” [1]. Although all do not accept this definition, it has helped to guide an expert consensus opinion that most forms of hydrocephalus can be classified according to an identifiable anatomical site of CSF flow obstruction or impairment [2]. There is currently no definitive cure. Most patients are managed by shunting using a silicone tube and valve system, where CSF is diverted from the cerebral ventricles to another body site [3]. Treatment by shunting is associated with frequent complications, particularly obstruction and infection in young infants, which add to morbidity and mortality. In a large population-based analysis in California (1990–2000), the cumulative 5-year complication rate was approximately 48 % in children and 27 % in adults [4]. A recent pediatric study showed a 33 % shunt failure in 3 years [5]. Surgical alternatives to ventriculo-peritoneal shunting, such as endoscopic third ventriculostomy (ETV), have become popular. In controlled trials, ETV has reasonable efficacy in pediatric hydrocephalus particularly for aqueduct stenosis [6, 7]. ETV requires more robust evidence for routine use in adult normal pressure hydrocephalus (NPH) [8]. Lumboperitoneal shunting also shows some promise in NPH [9].

Because of the potential for complications, neurosurgeons may be hesitant to operate on young (especially preterm) infants with hydrocephalus. However, enlarging ventricles and delayed shunting might be associated with progressive brain damage [10]. For more than six decades, researchers have been trying to develop nonsurgical means for management of hydrocephalus. Three main categories of treatment have been explored in animal models and, in some situations, hydrocephalic patients. These include reducing CSF production, reducing the inflammatory and fibrotic processes that occur following brain hemorrhage or meningitis, and “protection” of brain cells and axons after ventriculomegaly has developed. The latter is envisioned as a supplemental intervention that would allow a safe delay in surgical shunting of hydrocephalus.

The purpose of this review is to summarize and evaluate research concerning pharmacological therapies for hydrocephalus. The goal is to be comprehensive with an explanation of the rationale in animal experiments and a careful consideration of the earliest and the most definitive clinical trials. The review is organized in respect to the pathophysiologic factors involved in the development of hydrocephalus, specifically CSF production, CSF pathway obstruction (inflammation, fibrosis), and brain damage. Because some aspects of the pathogenesis of hydrocephalic brain damage are very similar to those that occur in cerebral ischemia (stroke) and brain trauma, evaluation of preclinical research should be similarly robust. Therefore, the quality of preclinical research studies related to brain protection will be judged using guidelines originally proposed by the Stroke Therapy Academic Industry Roundtable group [11, 12] and adopted by brain trauma investigators [13].

## Methods

The senior author beginning c2000 has collected literature related to this topic. This was supplemented by structured searches in PubMed and GoogleScholar using the main term “hydrocephalus” along with key words “anti-inflammatory, diuretic, drug therapy, drug effects, fibrinolysis, inhibitor, neuroprotective, osmotic, pharmacology, randomized, steroid”. Titles and abstracts were reviewed to ensure that the main topic was hydrocephalus. There is considerable overlap with the literature concerning brain or subarachnoid hemorrhage; only papers related to CSF flow or ventricular enlargement are addressed here. Relevant manuscripts were read in detail, and additional publications that had not been identified in the search were sought from the bibliographies (especially pre-1960, a period when works are less comprehensively recorded in PubMed). There were no time limits imposed; historical context is considered important. Considering the paucity of modern randomized trials in humans, this review is not meant as a meta-analysis; there are no specific exclusion criteria. In general, the sections are organized to separate the historical and experimental background from the pediatric and adult clinical studies, although this is not always possible. Treatments for shunt infection are not included. Detailed consideration of the pharmacodynamics of the drugs is beyond the scope of the review, although they will be mentioned when relevant to the interpretation of selected experiments. Some of the pharmacologic factors were described in a previous review [14]; details of the pharmacodynamics are beyond the scope of this review.

## Results

### Cerebrospinal fluid production and brain fluids

Readers are referred to other reviews for comprehensive considerations of choroid plexus function including CSF production [15–17], flow of brain fluids through intercellular and paravascular compartments [18–21], and CSF absorption [22, 23].

#### Osmotic agents and CSF pressure

One of the first pharmacologic attempts to manage hydrocephalus was by Marriott who, in 1924, reported that the diuretic agent theobromin sodio salicylate stabilized the head size of six infants with progressive hydrocephalus [24]. In the 1950s and early 1960s, when shunt therapy was emerging, drug therapies for hydrocephalus were directed at reducing CSF production or dehydrating the brain by diuresis. Typically, an appropriate physiological response in normal animals was sufficient rationale for testing of a drug in hydrocephalic humans. An excellent pharmacologic review of these agents was previously published [14].

Isosorbide, a dihydric alcohol derived from sorbitol that functions as an osmotic diuretic, is the best studied in hydrocephalus. Oral isosorbide transiently reduced the CSF pressure in healthy adult dogs [25]. A similar reduction in ventricular CSF pressure was demonstrated in 14 hydrocephalic children, although a rebound above the baseline was reported in 5/10 children who received multiple doses [26]. In what were described as preliminary experiments, 45 hydrocephalic patients (newborn to 62 years, median age 1 month) received 1–3 g/kg orally up to six times per day for 1–54 days (median 4 days) and were monitored for benefits defined as reduced CSF pressure or head size; 53/97 treatment episodes had some benefit and 26/97 had adverse effects [27–29]. Subsequently, Lorber conducted uncontrolled trials of isosorbide in infants with many forms of hydrocephalus; he eventually concluded that it was an effective adjunct for temporarily controlling intracranial pressure (ICP) prior to shunting, but not a replacement for surgery except perhaps in a minority of infants with myelomeningocele [30–33]. A controlled study of children with myelomeningocele two decades later compared 14 treated with isosorbide and 17 managed without the drug. Although isosorbide allowed delay of the surgical closure, there was no difference in need for shunting; furthermore, side effects led the authors to conclude that it has no role in management of these children [34].

Glycerol, a trivalent sugar alcohol, was known as a diuretic agent since the early 1900s [35]. As an oral osmotic agent, it was shown to reduce intracranial pressure in adults with brain tumors and was suggested as a possible agent for managing hydrocephalus [36]. However, a few small, uncontrolled trials did not support its use. Glycerol had no effect in four premature infants with posthemorrhagic hydrocephalus [37] and did not alleviate hydrocephalus in adults with metastatic brain cancer [38]. Although not a therapeutic effect, NPH patients who displayed an increase in cerebral blood flow (CBF) after glycerol administration tended to have a favorable response to shunt surgery [39]. Other sugar alcohols, mannitol and erythritol, transiently decreased ICP in adult hydrocephalic dogs following intravenous administration [40]. Although used widely for temporary management of elevated ICP in situations of brain swelling, mannitol has seldom been used except in adults with acute hydrocephalus caused by intracerebral hemorrhage [41]. In two chronically hydrocephalic children mannitol or urea transiently reduced ICP, however there was a rebound above baseline pressure [26].

#### Interference with CSF production

Because CSF dynamics are affected in obstructive forms of hydrocephalus, beginning in the 1950s and 1960s researchers have sought to treat hydrocephalus by reducing CSF production.

#### Acetazolamide

Acetazolamide, a carbonic anhydrase inhibitor, decreases CSF flow and ICP in rabbits and cats [42–44]. This was initially thought to be a diuretic and natriuretic effect through inhibition of carbonic anhydrase in the kidneys. However, later it was determined that choroid plexus has high levels of carbonic anhydrase and acetazolamide causes direct inhibition of CSF production. More recently, acetazolamide was also shown to inhibit aquaporin-mediated water conductance through several molecular pathways [45]. Acetazolamide was shown to reduce the lateral ventricle size of young adult rats, 1 day following injection of thrombin into the ventricle [46, 47]; however, ventricular enlargement was minimal in controls and the survival time too brief to be meaningful. Veterinary use of acetazolamide in hydrocephalus dogs is not beneficial [48].

#### Acetazolamide in hydrocephalic children

In trials beginning 1956, Elvidge and coworkers found that oral acetazolamide improved the clinical condition of a hydrocephalic child who was suffering from numerous shunt complications; vasopressin had no beneficial effect [49]. Benefit was also reported anecdotally and in small trials by others [50–55]. However, detailed physiologic studies on humans showed inconsistent reductions in CSF production [56]. By the late 1960s, it had become apparent that the therapeutic effect of acetazolamide in hydrocephalic children was negligible [57, 58].

#### Acetazolamide in hydrocephalic adults

Despite failed use in children, acetazolamide was proposed as an alternative to shunting for adults with NPH [59]. Although there was no persisting effect on ICP, the transient reduction in ICP following an acetazolamide bolus was shown to be predictive of good response to shunting in NPH patients [60]. Normal people have a transient increase in CBF (measured by first-pass radionuclide angiography) in response to a bolus; people with NPH have an impaired cerebrovascular response [61] and a blunted response was reported to be predictive of good response to shunting [62]. The poor increase in CBF following acetazolamide is thought to indicate a low capacity for vasodilation in the hydrocephalic brain, possibly due to compression of blood vessels [63]. In 5/8 NPH patients, administration of oral acetazolamide (125–375 mg/day) was associated with a decrease in the periventricular hyperintensities seen on magnetic resonance (MR) imaging [64] and an increase in CBF measured using MR arterial spin labeling [65]. There remains limited interest in the use of acetazolamide for treatment of idiopathic intracranial hypertension [66, 67].

#### Furosemide/acetazolamide combination in hydrocephalic children

Furosemide is a diuretic agent that inhibits the Na–K–2Cl symporter located in the distal tubules of kidney. Furosemide has minimal effect on CSF formation in healthy cats and dogs [44, 68], but CSF formation was reduced in preterm rabbits and in adult rabbits, albeit much less potently than acetazolamide [69, 70]. Vinas mentioned use of furosemide in management of a hydrocephalic child [71]. Chaplin and coworkers claimed to have arrested the progression of hydrocephalus in 4/7 infants with posthemorrhagic hydrocephalus by repeated CSF drainage and administration of oral acetazolamide and furosemide [72]. Shinnar et al. [73] gave the drug combination to 49 hydrocephalic children; they reported a 57 % success rate (avoidance of shunt) in non-myelomeningocele patients but only 26 % success in infants with myelomeningocele. An uncontrolled trial conducted from 1982–86 also hinted at benefit [74, 75]. A randomized control trial of the acetazolamide/furosemide combination for premature infants with posthemorrhagic ventricular dilatation was conducted from 1992–96. Unfortunately it failed to show benefit [76–78]. This trial was criticized for its failure to prove a solid rationale and safety background before starting [79].

#### Other ion channel blockers

Other drugs that block ion channels have been tested in small numbers of hydrocephalic patients. Digoxin, an inhibitor of Na–K–ATPase, reduces CSF production in humans at doses that are not cardiotoxic [80]. Digoxin was reported to reduce CSF production in three hydrocephalic children [81], but in three hydrocephalic infants it failed to prevent enlargement of the head [82]. Triamterene is a diuretic drug that blocks the epithelial sodium channel (ENaC). Three adults (age 25–76 years) with chronic hydrocephalus had resolution of symptoms during a 3–12 week treatment with triamterene [83]. Antagonists to the transient receptor potential vanilloid 4 (TRPV4), a gated divalent cation channel highly expressed by choroid plexus [84], have also been proposed as a treatment for hydrocephalus [85], but there are no published data supporting the concept.

#### Glucocorticoids

Steroids, in particular the glucocorticoids, have been used for decades with variable benefit in a range of neurological disorders associated with raised ICP. In healthy rabbits, betamethasone reduced CSF production after 5 days [86]. In normal dogs and in dogs with kaolin-induced hydrocephalus, intravenous dexamethasone or methylprednisolone caused a 40 % reduction in CSF flow for approximately 6 h, in the absence of diuresis or changes in blood pressure [87, 88]. The effect is through specific binding to the mineralocorticoid receptor (NR3C2; nuclear receptor subfamily 3 group C member 2) on choroid plexus epithelial cells, with subsequent regulation of several functions [89]. In 13 shunted hydrocephalic children with symptomatic slit ventricle syndrome, dexamethasone was associated with temporary relief of symptoms, although 9/13 required subsequent surgical intervention [90]. In the context of hydrocephalus, corticosteroids have also been used as a possible way to reduce the fibrosis in the subarachnoid compartment (see below).

#### Choroid plexus destruction

Surgical ablation of the choroid plexus as a means to control hydrocephalus was proposed by Dandy [91]. Using modern surgical techniques in combination with endoscopic third ventriculostomy, Warf has shown that this approach can be successful in selected cases less than 1-year age [92]. Non-surgical means of destroying the choroid plexus have also been attempted. In the 1970s, several groups of investigators injected radioactive agents into the ventricles of cats and dogs with kaolin-induced hydrocephalus. Gold-198 in colloidal form caused necrosis of the choroid plexus and reduced CSF production [93–95]. Rhenium-188 [96] and technetium-99 m [97] were not effective in similar model systems. Intraventricular injection of radioactive chromic phosphate-phosphorus-32 into four hydrocephalic infants was reported to arrest head growth, but long-term results were not presented [98]. Likely due to the high potential for side effects, others have not pursued this approach.

Destruction of choroid plexus by ricin toxin-conjugated to antibodies that bind to the epithelial cells was tested in culture and was proposed as a means for managing hydrocephalus [99, 100]. However, there are no published experiments using animal models.

#### Summary of CSF modulation for managing hydrocephalus

Reduction of brain water with osmotic agents and inhibition of choroid plexus ion channels provide transient reductions in the ICP or CSF pressure of hydrocephalic children and adults. However, no lasting effects have been demonstrated in published clinical trials, although recent work in NPH suggests that there might be a role for acetazolamide at least as a test agent to predict response to shunting [101]. Considering that the half-life of acetazolamide is 4 h, divided dosing regimens might be needed to achieve optimal effectiveness. Conceivably, highly selective inhibition of choroid plexus ion channels that are not present in other epithelia (e.g. kidney) might be associated with fewer side effects and might work in situations where obstructions to CSF flow are only partial. It will likely be difficult to avoid toxicity in non-surgical ablation approaches.

### Cerebrospinal fluid pathway modulation

#### CSF pathway inflammation and fibrosis

One of the general features of hydrocephalus is a relative restriction of CSF movement (bulk flow or pulsatile movement) along its anatomical route [2]. Circumstances that can lead to restrictions include intraventricular hemorrhage, subarachnoid hemorrhage, or infection (e.g. meningitis); all are associated with secondary inflammation and fibrosis in the CSF pathways, especially the subarachnoid compartment [102]. In children, intraventricular hemorrhage and bacterial meningitis are associated with meningeal fibrosis, which obliterates the subarachnoid space [103, 104]. In adults with subarachnoid hemorrhage, inflammation occurs within the arachnoid villi during the first week and is followed by proliferation of arachnoid cap cells [105] and collagen production [106]. Enzymatic dissolution of the intraventricular or subarachnoid blood collections, interference with the inflammatory process, and interference with the production of extracellular matrix molecules are approaches that have been explored as ways to reduce the likelihood of developing hydrocephalus.

#### Enzymatic dissolution of intracranial blood collections in animals

Tissue plasminogen activator (tPA) is a serine proteinase that binds to fibrin in clotted blood and converts plasminogen to plasmin, thereby initiating fibrinolysis and dissolution of the blood clot. Urokinase (or urokinase-type plasminogen activator/uPA) is a proteolytic enzyme present in urine, blood, and extracellular matrix; it is also capable of activating plasminogen and it can digest extracellular matrix proteins [107]. Streptokinase is a bacterial enzyme capable of cleaving plasminogen, although it is less discriminate and can activate plasminogen that is not associated with blood clots [108]. Thrombolytic therapies for ischemic stroke were first pursued in the 1970s; intravascular recombinant tPA is an important intervention for some adults with early ischemic stroke [109]. However, tPA can have adverse effects in the brain parenchyma [110].

Experimental brain hemorrhage models have been used to determine if these agents can accelerate clot lysis and reduce the subsequent development of hydrocephalus. In dogs with experimental subarachnoid hemorrhage, intrathecal urokinase reduced fibrosis in the intracranial subarachnoid compartment at 3 weeks and 3 months [111]. The number of subarachnoid macrophages was greater following urokinase than in control dogs [112]. In dogs with experimental intraventricular hemorrhage (IVH), urokinase effectively lysed the blood clot in 3–6 days (vs. 38–65 days in controls); only 2/10 treated dogs (compared to 9/10 control dogs) developed progressive hydrocephalus [113]. Cats that were treated with an infusion of intrathecal tPA 1 day after the intracisternal blood injection did not develop hydrocephalus at 7 days [114]. Pigs with experimental IVH had more rapid resolution of the blood clot and reduction in the ventricle size at 1 week after treatment with tPA, although the control animals had reached comparable ventricle size by 6 weeks [115, 116]. In the collagenase model of intracerebral hemorrhage in young adult rats, infusion of urokinase or tPA into the lateral ventricle for 1 h beginning 3 h after collagenase was associated with reduced ventricle size measured by MRI after 3 days. However, only urokinase was associated with improved behavioral outcome [117].

#### Thrombolytic agents in children with posthemorrhagic hydrocephalus

Pediatric clinical trials in the 1990s initially showed some promise for prevention of post-hemorrhagic hydrocephalus through use of several thrombolytic agents. Premature infants with IVH (n = 18 vs. 39 controls, nonrandomized) were treated with intraventricular urokinase for 7 days; they were reported to have a lower requirement for shunting than historical controls (37 vs. 92 %) [118, 119]. In a pilot study, six premature infants with IVH, ventricular dilation, and head enlargement (>2 cm per week) despite diuretic therapy and serial lumbar punctures received intraventricular urokinase for 3 days. Although the intraventricular clot decreased in size and there were no apparent side effects, all infants required a shunt. The authors concluded that late initiation of urokinase was ineffective in this situation [120]. Following the observation that CSF plasminogen levels were low in premature infants with IVH [121], Whitelaw and coworkers administered intraventricular bolus of recombinant tPA to 22 infants with post-hemorrhagic hydrocephalus; among the 21 survivors, 12 did not require a shunt [122]. A phase 1 clinical trial then showed that 17/23 survivors did not require a CSF shunt [123]. However, a multicenter randomized trial of 70 infants showed that intraventricular tPA and CSF washing (drainage, irrigation, and fibrinolytic therapy; DRIFT) did not reduce shunt surgery or death and was potentially associated with secondary intraventricular hemorrhage [124]. Although the immediate results did not look favorable, a 2-year follow-up of surviving children from the DRIFT trial indicated that treatment was associated with a reduction of severe cognitive disability [125], but this did not correlate with any improvement in brain volume measured by MRI [126].

During this same period, nine preterm infants received intraventricular infusion of streptokinase for 12–72 h without major adverse events [127]. A randomized case-control trial conducted 1992–1994 included six infants who received intraventricular streptokinase for 4 days. Although fibrin degradation products were significantly elevated and blood clot reabsorption occurred after infusion, there was no difference in the number of children who required shunting (3/5 survivors in each group); the authors concluded that they could “not recommend fibrinolytic intraventricular infusion as a routine treatment” [128]. Another prospective case control trial of six treated and six untreated infants also led to the conclusion that streptokinase should not be used in this situation [129]. In 1999, the authors of an excellent review of the five small case series published to that time concluded, “While there is insufficient evidence to justify any claim that fibrinolytic therapy is safe and effective, there is also insufficient evidence to justify discarding this form of treatment as ineffective or unsafe” [130]. Systematic reviews of the randomized trials show no support for intraventricular streptokinase treatment after intraventricular hemorrhage in premature infants [131].

#### Thrombolytic agents in adults with posthemorrhagic hydrocephalus

Similar clinical approaches have been used with varied success in adults with hydrocephalus following IVH or subarachnoid hemorrhage. Among six adults with severe IVH who received intraventricular urokinase for 7 days, 5/6 had a good or excellent outcome, while only two required shunting for hydrocephalus [132]. A small randomized trial of adults with IVH showed a lower mortality and slightly lower likelihood of need for shunt (3/9 vs. 2/4), but a high incidence of very poor neurological outcome among survivors (7/10) [133]. A retrospective study of 14 adults with spontaneous IVH and hydrocephalus treated with intraventricular urokinase from 2002–2005, suggested that the therapy was safe; in comparison to historical controls, it seemed to be associated with reduced mortality and reduced incidence of ventricular drain obstruction [134]. Wang advocated for intrathecal administration of urokinase to treat acute hydrocephalus in adults [135]. A pilot experiment in adults with IVH showed that intraventricular tPA was associated with reduction of intraventricular blood and normalization of ventricular size within 1–2 days [136]. In an uncontrolled trial, adults with intracerebral hemorrhage and hydrocephalus exhibited rapid resolution of the hematoma when tPA was infused into the ventricle; compared to the historical controls, tPA infusion combined with lumbar drainage seemed to reduce the need for CSF shunting (33 vs. 3 %) [137, 138]. High dose tPA (4 mg tPA every 12 h to a maximum cumulative dose of 20 mg tPA) was associated with more rapid clot resolution from the lateral ventricles but not the third or fourth ventricles [139]. In 2007–2008, 41 subarachnoid hemorrhage patients received intraventricular tPA for 5–7 days. In comparison to historical controls, tPA recipients had fewer days in severe vasospasm and the incidence of hydrocephalus requiring shunt was also lower (18 vs. 43 %) [140]. A randomized double blind trial testing the value of intraventricular tPA for adults with IVH was underway in 2015 [141]; interim analysis showed that the rate of rebleeding was not a major risk [142]. Unfortunately, the need for a shunt to treat hydrocephalus is not one of the planned outcome criteria.

#### Summary of blood clot lysis for hydrocephalus

In general, animal and human studies (both pediatric and adult) show that enzymatic blood clot lysis accelerates the resolution of the blood collection, but offers no long-term protection against the development of hydrocephalus [143, 144]. Authors of a systematic review concluded “intraventricular thrombolytic agents including tPA, urokinase, or streptokinase are not recommended as methods to reduce the need for shunt placement in premature infants with [posthemorrhagic hydrocephalus” (high clinical certainty) [145]. Similarly, a meta-analysis of trials on adult patients with aneurysmal IVH concluded “intraventricular rt-PA has no significant efficacy on the long-term functional recovery” [146]. A more recent trial found that intraventricular tPA had no effect on the need for CSF shunt dependency or the functional outcome of adults with endovascular-treated aneurysmal SAH [147]. Recent data suggest that the sudden release of blood breakdown products by fibrinolysis might fail to protect against hydrocephalus because inflammation is increased by rapid blood clot lysis [148]. Nevertheless, there remains the possibility that tPA in children with posthemorrhagic hydrocephalus might offer some long-term benefits unrelated to the development of ventriculomegaly.

#### Experimental interference with inflammation in the meninges

There is a clear association between inflammation in the CSF pathways and subsequent development of hydrocephalus. Therefore, anti-inflammatory agents have been tested experimentally to prevent post-meningitis and post-hemorrhagic hydrocephalus. Rabbits with meningitis caused by *S. pneumoniae* develop increased resistance to CSF outflow. If they are treated with methylprednisolone within 1 day after the bacterial inoculation, CSF outflow is normalized, likely due to decreased inflammation [149]. Adult rabbits develop hydrocephalus following intraventricular blood injections repeated over a 2-week period. Those that received concurrent intramuscular methylprednisolone had significantly reduced severity of hydrocephalus, but intraventricular methylprednisolone caused ventricular enlargement in 35 % of rabbits [150]. A detailed study of post-hemorrhage fibrosis in the subarachnoid compartment of dogs with autologous blood injection into the cisterna magna showed that intrathecal dexamethasone did not significantly alter the fibrosis at 3 weeks or 3 months [151]; the ventricle size was not measured in these dogs.

#### Anti-inflammatory agents in humans with meningitis

In humans, high quality randomized trials of corticosteroid therapy for acute bacterial meningitis show significantly reduced hearing loss and neurological sequelae, although there are no specific data concerning development of hydrocephalus [152]. A recent retrospective study of adults with bacterial meningitis suggests that increased brain ventricle size in the acute phase of bacterial meningitis is associated with increased mortality [153]. A meta-analysis of clinical trials using corticosteroids to treat tuberculosis meningitis indicated a marginal benefit with reduction of death and disabling residual neurological deficit amongst survivors [154], but the likelihood of developing hydrocephalus was not changed [155, 156]. However, a recent randomized study of children with tuberculous meningitis suggested that high dose prednisolone (4 mg/kg/day over 4 weeks vs. 2 mg/kg/day) might be associated with increased risk of hydrocephalus [157]. A rare congenital autoimmune disease called chronic infantile neurologic, cutaneous, articular (CINCA) syndrome is characterized by neonatal-onset chronic meningitis; a single child who developed hydrocephalus was successfully managed with anakinra, a recombinant interleukin-1-receptor antagonist [158].

#### Role of transforming growth factor beta in the development of subarachnoid fibrosis

Considering the marginal successes of blood lysis and anti-inflammatory agents for managing hydrocephalus, a more targeted molecular approach seems worth considering. Transforming growth factor beta (TGF-β) is a growth factor released from platelets at sites of blood clotting whereupon it regulates proliferation of fibroblasts (and other cell types), as well as the synthesis of extracellular matrix proteins. In 1994, high levels of TGF-β1 in the CSF of adults with subarachnoid hemorrhage were shown to be associated with enlargement of the cerebral ventricles [159, 160]. A similar association was later shown in premature infants with IVH [161, 162]. However, it must be noted that recent data obtained from a CSF assay study failed to support a critical role for TGF β1 and TGF β2 in the development of posthemorrhagic hydrocephalus in adults [163].

In neonatal rats with intraventricular blood injection, TGF-β1, TGF-β2, and TGF-β3 are increased, although only the latter was associated with enlarged ventricles and only TGF-β1 was restricted to the meninges. This was accompanied by deposition of fibronectin, laminin, and vitronectin in brain tissue [164]. In animal experiments, injection of TGF-β1 into the subarachnoid compartment of 10-day old mice caused hydrocephalus [165, 166] as a consequence of progressive fibrosis in the leptomeninges [167]. Intracerebroventricular infusion of recombinant human hepatocyte growth factor, which antagonizes the fibrosis-inducing effect of TGF-β1, for 7 or 14 days, improved the memory, ventricle size, CSF flow, and meningeal fibrosis in mice with TGF-β1 injection-induced hydrocephalus [168].

Transgenic mice with TGF-β1 overexpression linked to the glial fibrillary acidic protein (GFAP) promoter in astrocytes develop hydrocephalus with no obstructions in the ventricular system [169, 170]. These mice have increased cellularity and obstruction to CSF flow in the subarachnoid compartment, and they develop enlarged ventricles beginning at postgestation day 15 [171–173].

Interference with the TGF-β1 pathway has been explored as a means to treat or prevent experimental hydrocephalus. Seven-day-old rats with post-IVH hydrocephalus were randomized to receive the drugs pirfenidone or losartan, which have been shown to reduce TGF-β expression and decrease inflammatory fibrosis in lungs and other organs. After 14 days treatment, neither drug had a beneficial effect on ventricle size or behavior [174]. Infusion of thrombin into the subarachnoid compartment of adult rats mimics some of the changes that occur after subarachnoid hemorrhage, including increased expression of TGF-β1 and meningeal collagen accumulation. Administration of the TGF-β1 inhibitor SB-431542 into the cisterna magna prior to thrombin injection blocked the collagen accumulation at 10 and 20 days [175].

Three-week old rats received subarachnoid injections of kaolin with or without infusions of decorin, a small proteoglycan that can bind TGF-β1. There was good evidence that the decorin-treated rats had reduced inflammation and fibrosis in the subarachnoid compartment [176]. However, the fact that treatment began before the onset of hydrocephalus and the large number of exclusions from the decorin group makes this an interesting proof-of-principle only. A follow-up experiment by the same group is planned with delayed (1 week) initiation of decorin treatment (personal communication; Dr. P. McAllister September 2015). Another group used a subarachnoid model in 6-week-old rats; the rats received intracisternal injection of decorin immediately before blood injection followed by intracerebroventricular injection of decorin 10 days later. The treated rats convincingly had inhibited up-regulation of TGF-β1, reduced collagen, reduced ventricle size, and improved cognitive outcome [177].

The TGF-β and Wnt signaling pathways converge through the Smad/YAP complex, which translocates to the nucleus to activate various genes related to fibrosis [178]. Ventricle size and reactive astroglial changes were measured in adult rats following injection of kaolin into the lateral ventricle. If sFRP-1, a Wnt inhibitor, was added to the kaolin, the ventricle enlargement was less severe and the GFAP expression was reduced in periventricular regions of unspecified location [179]. Although not measured directly, inhibition of the Wnt signaling cascade might alleviate development of hydrocephalus by reducing fibrosis.

Young adult mice with kaolin-induced hydrocephalus were maintained on tap water or deuterium water beginning 7 days prior to kaolin. The stated rationale was previous evidence that deuterium could inhibit cell proliferation and fibrosis in vitro. After 4 weeks, the treated rats had ventricles of normal size, reduced collagen on extracted tissue sections, and reduced TGF-β1 immunoreactivity in posterior fossa compared to untreated hydrocephalic rats [180]. This demonstration of a seemingly miraculous treatment had several shortcomings; the most important being the failure to demonstrate that kaolin was actually delivered into the subarachnoid compartment. Finally, 7-day rats with periventricular hemorrhage induced by intracerebral injection of collagenase received the TGF receptor 1 inhibitor SD208 daily for 3 days, beginning 1 h or 3 days after collagenase injection. SD208 reduced the adverse behavioral effects, weight loss, and associated brain injury [181]. However, considering that the ventricular enlargement was unilateral and apparently due to brain atrophy, this experiment should not be considered evidence for treatment of hydrocephalus.

In summary, antagonism of the TGF-β1 pathway has excellent rationale for prevention of post-hemorrhagic or post-meningitis hydrocephalus, but more preclinical experimentation is required.

#### Deferoxamine for preventing fibrosis

Deferoxamine is a chelating agent that binds free ferric iron (Fe^3+^). It is used effectively in a variety of diseases associated with blood breakdown; it can prevent fibrosis by attenuating oxidative stress and the subsequent inflammatory cascade [182]. There are promising experimental data for treatment of intracerebral hemorrhage [183]. Ventricular enlargement was studied after fluid percussion brain injury in adult rats. Deferoxamine was administered beginning 2 h after injury and the brains were studied at 1 day. Deferoxamine was associated with normalized ventricle size and upregulation of heme oxygenase 1 [184]. In another lab, young adult rats received unilateral injection of autologous whole blood or ferric chloride (FeCl_3_) into the lateral ventricle. Intraperitoneal deferoxamine was administered beginning immediately afterward and then for 7 days; the brains were examined at 1 or 4 weeks. Blood or FeCl_3_ caused enlarged ventricles and deferoxamine treatment prevented ventricle enlargement [185, 186]. The authors discussed neuroprotective properties and anti-fibrotic properties of the agent, but did not directly study the meningeal compartment.

#### Reversing accumulation of extracellular matrix molecules in the subarachnoid compartment

As with any wound healing processes, hyaluronic acid and a variety of proteoglycans are produced in the first few days after a hemorrhagic or inflammatory process. Several weeks are necessary before collagen, which is more permanent, is produced [187, 188]. These fibrosis-associated molecules seemed a reasonable target, albeit a late one, for modulation in hydrocephalus. A variety of purified enzymes, including the matrix metalloproteinases (MMP), are capable of degrading a variety of extracellular matrix molecules.

In a rabbit model of IVH, ventricular enlargement occurs. Single intracerebroventricular injection of chondroitinase ABC, which digests a variety of proteoglycans, did not reduce the ventriculomegaly, although it should be noted that the subarachnoid compartment was not studied directly [189].

In a few small case series, animal-derived hyaluronidase was administered intrathecally on a weekly basis for several weeks to children with tuberculous meningitis and hydrocephalus. One of the earliest trials suggested that hyaluronidase was superior to shunt surgery [190]. A later trial showed improved level of consciousness but no overall improvement in comparison to CSF shunting [191]. One report claimed that hyaluronidase offered mild transient benefit in three children with myelomeningocele [192]. However, the largest published trial showed that hyaluronidase was associated with reduced intracranial pressure but no change in ventricle size in 12/17 children with tuberculosis-associated hydrocephalus [193]; it is considered of historical interest only [194].

The MMP are a family of diverse enzymes capable of degrading a variety of extracellular matrix molecules. In experimental hydrocephalus, adult rats had elevated MMP9 in the CSF after intraventricular kaolin injection [195] while transgenic mice with TGF-β overexpression have reduced MMP9 mRNA and activity [196]. MMPs were assayed in the CSF of 13 premature children with posthemorrhagic hydrocephalus, 10 infants with non-hemorrhagic hydrocephalus, and 10 controls. Pro-MMP9 was found to be elevated in the CSF in 9/13 infants that did not require a shunt and the authors speculated that modulation of MMP9 activity might be a therapeutic strategy worth testing [197, 198]. However, inspection of their data shows that two children with very high levels skewed the MMP9 level.

### Cerebral blood flow and pulsation in hydrocephalus

Diminished regional blood flow, particularly in the cerebral white matter, is well documented in pediatric and adult hydrocephalus [199, 200]. It is not entirely clear if this is a purely mechanical phenomenon that results from distorted or compressed blood vessels, or if there is a dysregulation due to altered innervation or local cell interactions. Regardless of the cause, hypoxia likely contributes to the pathogenesis of axon damage [201, 202]. Many adults with NPH have arterial hypertension and cerebrovascular disease [203, 204]. These epidemiologic data suggest that pre-existing vascular disease can aggravate the brain dysfunction associated with hydrocephalus or that the abnormal blood pressure can contribute to the development of ventriculomegaly. The role of vascular pulsations as mechanical contributor to ventricular enlargement is currently of great interest [205, 206]. A seldom-cited case report of an infant with hydrocephalus suggested that bilateral common carotid artery ligation was associated with reduced intracranial pulsation and stabilization of head size [207].

#### Vasoactive drugs in experimental hydrocephalus

Using the 3-week rat model of kaolin-induced hydrocephalus we demonstrated several aspects of the axonal damage that suggested a possible role for activation of proteolytic enzymes by intracellular calcium [208]. Based on that information, hydrocephalic rats were treated by continuous administration of the calcium channel blocker nimodipine for 2 weeks, beginning 2 weeks after induction of hydrocephalus (i.e. beginning at 5 weeks age). Nimodipine treatment prevented the declines in motor and cognitive behavior that were observed in untreated control rats, and although the degree of ventricular enlargement was equal, the corpus callosum was thicker in the treated rats [209]. We later showed that Mg^++^ (delivered as MgSO_4_), a Ca^++^ competitor, had a similar but weaker protective effect [210]. However, when nimodipine or Mg^++^ was administered to 1-week rats, there was no protective effect and the side effects were more substantial [211]. Recent experiments from our lab showed that both agents, at the doses that were protective, have significant vasoactive effects in rats; these include decreased systemic arterial blood pressure, decreased cerebral perfusion and intracerebral pulse pressure, but no change in CBF (unpublished work; Shulyakov and Del Bigio, in preparation). We went on to test MgSO_4_ in a ferret model of hydrocephalus [212]; two-week ferrets received kaolin injections and beginning 2 weeks later received MgSO4 by parenteral injections. Although they had significant side effects (lethargy), there was no evidence for protection at the behavioral, structural, or biochemical level (Di Curzio, Turner-Brannen, Mao, Del Bigio; manuscript submitted). We are planning a ferret experiment with nimodipine is planned to determine if the protection is reproducible in a gyrencephalic animal with hydrocephalus.

#### Vasoactive drugs in humans with hydrocephalus

One group treated 18 hydrocephalic infants with oral isosorbide dinitrate, a nitrate vasodilator (8 mg/kg/day; for 1–25 months, median 3 months) [213]; the authors seem to have mistakenly equated this drug with the osmotic agent isosorbide. They considered the treatment useful because only 2/10 posthemorrhagic cases required shunting (versus 5/5 myelomeningocele cases).

Eight adults with NPH underwent blood pressure monitoring during administration of the calcium channel blocker nimodipine, which is commonly used for treatment of hypertension. Four hours after nimodipine, the mean arterial blood pressure was lower, the ICP was slightly higher, and overall there was no change in cerebral perfusion pressure (estimated from the arterio-venous oxygen difference) [214]. The antihypertensive agent ketanserin was administered to eight patients with NPH as a single bolus; it caused a decrease in blood pressure without altering ICP of CBF [215]. These studies prove nothing in the context of managing hydrocephalus, although it does suggest that nimodipine should be safe.

Greitz suggested that selective venous constriction, for example using dihydroergotamine, might modulate the intravascular dynamics, although it should be noted that his logic was not entirely clear (“Venous constriction would result in dilatation of the venous side of the capillary, decrease of the microvascular resistance, opening up of the microcirculation and reduced intracranial pressure”) [216]. This approach has never been tested experimentally. Dihydroergotamine causes contraction of isolated human cortical arteries and veins [217], but it has no effect on CBF in normal adults [218]. It can reduce intracranial pressure in patients with traumatic brain injury [219], while it may be associated with increased intracranial pressure in brain tumor patients, possibly due to loss of autoregulation [220]. Based on these contradictory data, pursuing dihydroergotamine as a therapy for hydrocephalus seems unwise.

#### Vascular endothelial growth factor (VEGF) in hydrocephalus

VEGF is one of the best-known promoters of angiogenesis and vascular survival. It also plays a role in vascular permeability factor, and it may also provide direct trophic support to neurons [221]. Because of the known vascular and CBF alterations in hydrocephalic brains, VEGF is a logical target for investigation. VEGF function is mediated through specific receptors in the brain [222]. In a dog model of chronic hydrocephalus, significant decreases in frontal cortex VEGFR-2 were reported; VEGF was not altered in tissue, but it increased in the CSF [223]. In the same dogs, VEGFR-2 increased in the hippocampus; this corresponded to increased blood vessel density, which was not seen in frontal cerebrum [224]. This is of interest because typically the hippocampus sustains only minimal direct damage in hydrocephalic brains (although there is considerable damage to the connecting pathways) [225]. The authors suggested that modulation of VEGF and its receptor might be a useful therapy for the chronic brain hypoxia that occurs in hydrocephalus [226]. Moderate arm exercise in adults with chronic hydrocephalus was associated with elevated VEGF in the CSF [227].

In contradiction to the potential protective function of VEGF, another research group reported increased CSF levels of VEGF in children and young adults with hydrocephalus. They then infused VEGF into the cerebral ventricles of non-hydrocephalic rats and found that the ventricles were enlarged. Simultaneous infusion of bevacizumab, an antagonist of VEGF, prevented this enlargement [228]. The authors devised an extremely complicated postulate in which the VEGF interacts with VEGFR-2 on ependymal cells leading to dysfunction of fluid regulation and ultimately to hydrocephalus [229].

Thus, we have a situation where one group of investigators suggests that VEGF/VEGFR-2 alterations are secondary to hydrocephalus and represent a beneficial adaptation to chronic hypoxia, while another group claims that VEGF causes ventriculomegaly. This is a subject that needs clarification before drug interventions are tested in humans.

### Brain protection in hydrocephalus

Hydrocephalus is associated with gradual destruction of periventricular white matter leading to a disconnection syndrome. In the circumstance of the immature brain, hydrocephalus also interferes with developmental processes including cell genesis and myelination [201, 230]. In the situation of progressive ventricular enlargement wherein shunt therapy is likely necessary, drugs might be used to protect the brain temporarily prior to shunting. Considering the overlap in pathophysiology between hydrocephalus, stroke, and trauma, it is anticipated that candidates from the latter two diseases should be considered for protecting the hydrocephalic brain. In neonatal hypoxic-ischemic brain injury, a variety of potential early therapeutics have broadly been categorized into anti-oxidative, anti-inflammatory, anti-apoptotic, and anti-excitotoxic mechanisms, with melatonin and erythropoietin covering multiple mechanisms [231]. Additional file 1: Table 1 summarizes the experiments conducted to date in hydrocephalic animals including the rationale, strengths, and weaknesses.

#### Anti-inflammatory interventions and microglia

Microglial and astroglial activation occurs in animals and humans with hydrocephalus [232, 233]. Microglial response is reported to contribute to brain damage in many disorders [234], although microglial activation might also have beneficial effects [235, 236]. Nevertheless, until the damage-benefit balance related to microglia is settled, anti-inflammatory interventions could be considered in the treatment of hydrocephalus.

Minocycline is a semi-synthetic tetracycline derivative with anti-inflammatory properties. It appears to be protective in a range of animal neurological disorders as well as human stroke [237, 238]. Intraperitoneal administration of minocycline to H-Tx rats with congenital hydrocephalus from postnatal day 15 to day 21 was associated with increased cortical thickness (ventricle size was not measured) and reduced cerebral immunolabeling for Iba-1 (a microglial marker) and GFAP (an astrocyte marker) [239]. Young adult rats with kaolin-induced hydrocephalus were given minocycline from day 15 to day 21 after kaolin. Treated rats had reduced progression of the ventricle size and reduced Iba-1 and GFAP in the brain [240]. In 7-day-old rats that had intracerebral hemorrhage induced with collagenase injection, minocycline treatment administered daily for 5 days beginning immediately was also associated with improved behavioral outcome and less severe ventricular enlargement at 28 days [241]. Although minocycline seems promising, the main side effect of tooth discoloration must be considered in the pediatric population [242].

Transgenic mice with hydrocephalus secondary to TGF-β overexpression were administered the non-steroidal anti-inflammatory agents ibuprofen or pioglitazone, a peroxisome proliferator-activated receptor-γ agonist, from 2 to 4 months age. Although both suppressed activation of microglia (based upon cellular hypertrophy), neither drug had an effect on thioflavin S staining of amyloid in cerebral blood vessels and pioglitazone aggravated the ventriculomegaly (ventricle size was not measured in the ibuprofen recipients) [243]. It is not clear, however, that thioflavin S staining is a good outcome measure in young rats.

Adult rabbits were treated 1 day after intracisternal kaolin injection with a single dose of infliximab, a monoclonal antibody that binds to tumor necrosis factor-alpha (TNF), which is an important mediator of inflammatory responses that is produced by microglia. After 2 weeks, blood and CSF levels of TNF were reduced in treated rabbits, although ICP was not different between the groups. After sacrifice the only histological evaluation was terminal deoxynucleotidyl transferase-mediated dUTP nick end labeling (TUNEL) in the optic disc, optic radiation, and the lateral geniculate body; treated rabbits had fewer labeled cells with microglial morphology in the two latter locations [244]. Unfortunately, this experiment is very difficult to interpret because the ventricle size was not measured at any time and the significance of microglial death in the optic pathway is of unknown significance.

#### Antioxidative interventions

Numerous animal experiments and investigations of human CSF indicate that hydrocephalus is associated with oxidative and nitrosylative cell responses and damage, likely secondary to the chronic hypoxia [245, 246]. In 3-week rats with kaolin-induced hydrocephalus, epigallocatechin gallate derived from green tea and N-acetylcystein [sic] were both shown to reduce brain tissue malondialdehyde, one of the end products of lipid peroxidation [247, 248]. However, the biochemical assay was the sole outcome parameter (ventricle size was not measured) so the value of these interventions is unclear. Two-week old rats received daily melatonin injections for 4 weeks beginning immediately after intracisternal kaolin injection. Nitric oxide content was not significantly changed and glutathione was almost normalized in the choroid plexus of hydrocephalic animals [249]. However, ventricle size was assessed only qualitatively by inspection of the midsagittal slice, and the histologic interpretation of the choroid plexus is not convincing. Beginning 2 weeks after kaolin injection into 3-week-old rats, we administered an oral mixture of antioxidants (alpha-tocopherol, L-ascorbic acid, coenzyme Q10, reduced glutathione, and reduced lipoic acid) for 2 weeks. We detected no therapeutic effect on the analysis of behavior, brain structure, or biochemical content; of uncertain significance, we observed that the canola oil carrier normalized anti-oxidant capacity of the brain tissue [250]. Catechin polyphenols present in the leaves of *Camellia sinensis* are potent antioxidants. Seven-day-old rats were injected with kaolin and received extracts of *C. sinensis* daily beginning 2 days later. Although there was no behavioral protection and the ventricle size was not measured, treated rats had thicker corpus callosum at 10 days age (but not 21 days age) and had a significant reduction in the reactive astrocytes [251]. Using a similar protocol, the same group is currently doing a study with edaravone (30 mg/kg; Luiza da Silva Lopes, unpublished work), which is a free radical scavenger that appears to have some benefit in adults with acute ischemic stroke [252]. Adult rats received intraventricular injection of autologous whole blood; those that received simultaneous edaravone had less brain edema and less tissue malondialdehyde on day 1 and improved memory on day 28 [253].

#### Neuron and axon protection

Periventricular axons sustain most of the damage in hydrocephalic brains with secondary retrograde degenerative changes in some neuron populations [201, 230]. Memantine, a non-competitive NMDA receptor antagonist, protects neurons and axons in a variety of animal models [254]. One day after kaolin injection, 3-week old rats began receiving memantine daily for 2 weeks. Low resolution MRI verified the presence of hydrocephalus at the end of the study. Histologic counts indicated reduced neurons in the hippocampus of hydrocephalic rats, and partial recovery in the CA1 and CA2 (but not CA3) sectors of memantine-treated rats [255]. It should be noted that in other rodent hydrocephalus studies, hippocampal neurons are reported to be spared or show various secondary abnormalities due to deafferentation [256–258]. Memantine or an herbal medicine, Morinda citrifolia, were given to adult rabbits for 2 weeks beginning immediately after intracisternal kaolin injection; brain tissue around the fourth ventricle was analyzed by immunostaining for activated caspase-3 as a measure of cell death. The authors reported that memantine aggravated cell death while the herbal therapy was neutral with respect to hydrocephalus [259]. This experiment is flawed because the fourth ventricle size was not reported, the presence of kaolin in the immediate vicinity was not documented, and the caspase 3 immunostaining is not convincing. The lead author’s group is planning to test memantine in young hydrocephalic rats and measure behavioral, structural, and biochemical outcomes (experiment scheduled for early 2016).

Based on prior reports of axon protection in traumatic brain injury models, we treated 3-week-old hydrocephalic rats with tacrolimus, cyclosporine A, or calpain inhibitor I; there was no statistically significant protection in regard to behavior, brain structure or brain composition in any of the experiments [260]. In the same model system, we found no evidence for protection using the sodium channel-blocking agents mexiletine and riluzole [261].

#### Cerebral stimulants

Considerable experimental work [201] and many assays of human CSF [262] have showed that the monoamine neurotransmitter systems are disturbed in hydrocephalus. These correlate with some of the functional disturbances seen in hydrocephalic patients. A monoamine oxidase inhibitor, bifemelane, used as an antidepressant and “cerebral metabolic activator” tended to normalize norepinephrine and serotonin levels (albeit with wide variance) in the striatum and cerebral cortex of young adult rats with kaolin-induced hydrocephalus [263]. Several case reports of hydrocephalic patients treated with a variety of similar agents have been published. Methylphenidate, which is widely used for treatment of attention deficit hyperactivity disorder and which acts by blocking the dopamine and norepinephrine transporters, was administered to a single NPH patient (20 mg) in a double blind cross-over trial after she had been shunted; the drug as associated with improved cognitive performance and reduced apathy [264]. In another case report, a single NPH patient with depression responded well to twice-daily oral doses of 10 mg methylphenidate [265]. In two separate reports, two people with hydrocephalus and akinetic mutism (that persisted after shunt revision) responded well to bromocriptine and ephedrine [266, 267]; the former is a dopamine agonist and the latter is a sympathomimetic amine that increases the activity of norepinephrine. Finally, an unshunted 19 year-old with severe hydrocephalus and aggressive self-injurious behavior responded well to trazodone (200 mg/day) [268], which is an antidepressant of the serotonin antagonist and reuptake inhibitor class.

Although entirely anecdotal, this avenue of therapy seems open for further investigation.

#### Protection of the developing brain in early-onset hydrocephalus

A poorly studied aspect of hydrocephalus-associated brain damage is that due to disruption of the periventricular germinal matrix [269], which is the zone of proliferating cells that gives rise to neuron and glial precursors during human fetal life (or during the postnatal life of extremely premature infants).

In an elegant experiment, embryonic mice received injections of blood plasma, blood serum, or lysophosphatidic acid (LPA; which is a normal component of blood plasma) into the lateral ventricle on in utero day 13.5. These (but not red blood cells) caused severe hydrocephalus, which was progressive for at least 30 days after birth. The authors identified damage to the germinal cells along the ventricle wall and occlusion of the third ventricle as the cause of hydrocephalus. Pharmacological blockade with Ki16425, a receptor antagonist of LPA1 and LPA3, prevented the hydrocephalus [270]. The pathologic features in this mouse model resemble those in human fetal hydrocephalus caused by cryptic hemorrhage [271] and prematurity associated germinal matrix hemorrhage [272]. LPA from blood is postulated to disrupt the germinal zone. More experimental work is needed to determine if this can be developed into a practical clinical therapy for premature infants with intraventricular hemorrhage [273].

The H-Tx rat has inherited hydrocephalus that is caused by aqueduct stenosis, which develops around 18 days gestation [274]. The genetic cause of this partially penetrant disorder is not yet known [275]. Maternal administration (subcutaneous) of folic acid aggravates the incidence of hydrocephalus while a mixture of folinic acid and tetrahydrofolate significantly reduces the incidence of hydrocephalus. Various experiments led to the conclusion that folate imbalance is responsible for the defects that lead to this form of early-onset hydrocephalus [276].

#### Cell transplantation

Although cell transplantation is not, strictly speaking, a non-surgical intervention, it is mentioned here because it is not a shunt intervention. In one experiment, the serotoninergic innervation was destroyed by injection of the toxin 5,7-dehydroxytriptamine into brains of young adult rats, which were then rendered hydrocephalic by injection of kaolin. After 2 weeks, brain cells from 14-day gestation rat embryos were transplanted into the hippocampus; transplanted cells were detected by immunohistochemistry 4 weeks later. In the few animals studied, surviving cells were identified; the authors simply concluded that the hydrocephalic brain is capable of accepting transplanted cells [277]. Intracerebral transplantation of human umbilical cord mesenchymal stem cells was reported in a pair of experiments. Four-day-old rats received bilateral injections of maternal blood into the lateral ventricles, 2 days later MRI was used to evaluate the ventricle size, and then rats were randomized to receive intraventricular stem cells or fibroblasts or vehicle. By 32 days age, stem cell recipients (but not those in the other groups) had attenuation of the ventricle size as well as improved behavior, reduced brain cell death, and reduced reactive astroglial change. Reduced quantity of periventricular microglia and reduced levels of proinflammatory cytokines in the CSF and periventricular brain tissue led the authors to speculate that the mesenchymal stem cells reduced the hemorrhage-associated inflammation [278, 279], but this mechanism was not proved.

## Conclusions

Hydrocephalus can be modeled in a wide range of animal species by injection of blood or kaolin into the CSF pathway, or by genetic manipulation [280]. Many of the models reflect the pathogenesis of hydrocephalus in human disorders. Several pharmacologic interventions appear to alleviate experimental hydrocephalus over relatively short terms (i.e. weeks). Manipulation of water balance or CSF production by osmotic agents or ion channel antagonists also works transiently in humans. However, poorly controlled clinical studies and rare randomized trials show no overall benefit in the management of hydrocephalic patients. CBF response to acetazolamide challenge appears to have a role as a test for predicting shunt responsiveness in adults with NPH. There may be some opportunity for revisiting acetazolamide with proper dosing in accordance with its pharmacodynamics or development of agents that more selectively inhibit the choroid plexus.

Fibrinolytic agents that hasten the resolution of intraventricular or subarachnoid blood collections do not clearly prevent the development of hydrocephalus in infants or adults. A European survey conducted in 2010 indicated that there remains no consensus on the treatment of infants with post hemorrhagic hydrocephalus; only a small minority of neonatal care centers (6 %) was using fibrinolytic agents [281, 282]. Nevertheless, attenuation of the iron toxicity (e.g. with deferoxamine) remains a potential target for reduction of the inflammatory and fibrotic reactions that block the CSF pathways after hemorrhage or meningitis. Targeted molecular manipulation of the cellular mechanisms that lead to fibrosis in the leptomeninges (e.g. of TGF beta or pathways) might reduce obstruction to CSF flow through the subarachnoid compartment, thereby preventing the development of hydrocephalus. Once the reactive extracellular matrix molecules are established in the subarachnoid compartment, opportunities for digesting them seem limited.

Modulation of cerebral blood flow through vasoactive agents also seems sensible, but this work remains in the preclinical status. Use of cerebral stimulants to augment compromised neurotransmitter systems is wide open for investigation. Brain protection is also in the early preclinical stages. Minocycline, which acts through multiple molecular routes [283], shows early promise. Anti-oxidant therapies have typically been unsuccessful, or the experiments are limited by drug administration in a clinically irrelevant manner or by inadequate outcome analysis.

Perhaps surprising, some multifunctional agents that have progressed into clinical trials have not been tested in hydrocephalus. Erythropoietin is a multifunctional molecule that is neuroprotective through its receptor (EpoR) and has systemic anti-inflammatory and angiogenic effects that can reduce brain injury. Several randomized trials are underway to determine its effect in preterm infants [284]. A retrospective analysis of extremely low birth weight infants treated with erythropoietin from 1993 to 1998 indicated that hydrocephalus requiring shunting occurred in 4.5 % of treated survivors and 7.0 % of controls [285]. Other less well-studied agents (e.g. ginsenoside [286]) also deserve attention.

Brain protection has been the Holy Grail for thousands of stroke and trauma experiments, and has almost always failed in translation to the clinical setting [13, 287–291]. Those interested in hydrocephalus must take great care in the preclinical development of proposed therapies. Based upon recommendations from the stroke and brain trauma literature [11–13], we must design robust experiments including the following considerations: (a) the chosen animal model of hydrocephalus must have validity with respect to the human situation of interest (rodent models may be useful for screening but verification should be performed in a larger animal with gyrencephalic brain and voluminous white matter); (b) ventricle size should be measured before and after the intervention (preferably using in vivo imaging); (c) drug assignments should be randomized or organized to ensure that groups are comparable; (d) evaluations must be conducted by blinded investigators; (e) the primary outcome measures must be pre-determined; (f) outcome measures should include a combination of morphological, behavioral, and biochemical parameters; (g) drug administration should mimic the timing, delivery route, and dosage feasible in humans; careful attention must be paid to the pharmacokinetic properties of agents to be tested to ensure an optimal response; blood brain barrier permeability must also be considered because, in general, the blood brain barrier is intact in the hydrocephalic brain [292]; (h) drug administration should not involve pre-ventriculomegaly administration except as proof-of-principle (unless the agent is being used to prevent hydrocephalus in high risk situations); (i) statistical assessment of outcomes should use appropriate corrections for multiple comparisons; (j) candidate drugs should work in multiple species and in multiple laboratories before testing in humans. Unlike stroke and trauma in which most of the damage is initiated within the first few minutes, hydrocephalus is progressive; therefore, in the absence of a shunt intervention or in the circumstance of chronic static hydrocephalus, long-term outcomes may not be possible.

Despite over 5 decades research, no compelling non-surgical therapies have been developed for hydrocephalus. However, there remain several targets for prevention of hydrocephalus or as a complement to shunting. Ultimately, high quality preclinical experiments should be used to design clinical trials for premature neonates and adults in which ventricle size and need for shunting are included among the main outcome measures. Complex experiments testing augmented recovery or regeneration after shunting have not been attempted, but may be needed to address the clinical fact that some patients ultimately do need a shunt.

## Additional file

**Additional file 1. Table 1:** Summary of brain protection experiments in animal models of hydrocephalus including details of animal, age, drug intervention, outcome parameters, weaknesses, and overall strength of conclusions.

## Abbreviations

CA: cornu ammonis
CSF: cerebrospinal fluid
ETV: endoscopic third ventriculostomy
GFAP: glial fibrillary acidic protein
ICP: intracranial pressure
IVH: intraventricular hemorrhage
LPA: lysophosphatidic acid
MMP: matrix metalloproteinase
NPH: normal pressure hydrocephalus
TGF-β: transforming growth factor beta
TNF: tumor necrosis factor
tPA: tissue plasminogen activator
TUNEL: terminal deoxynucleotidyl transferase-mediated dUTP nick end labeling
VEGF: vascular endothelial growth factor
